# miR-155 regulates the proliferation and invasion of clear cell renal cell carcinoma cells by targeting E2F2

**DOI:** 10.18632/oncotarget.7951

**Published:** 2016-03-07

**Authors:** Yu Gao, Xin Ma, Yuanxin Yao, Hongzhao Li, Yang Fan, Yu Zhang, Chaofei Zhao, Lei Wang, Minghui Ma, Zhengwei Lei, Xu Zhang

**Affiliations:** ^1^ Department of Urology, Chinese PLA General Hospital/Chinese PLA Medical School, Beijing, 100853, P. R. China; ^2^ State Key Laboratory of Kidney Diseases, Chinese PLA General Hospital/Chinese PLA Medical School, Beijing, 100853, P. R. China

**Keywords:** miR-155, proliferation and invasion, E2F2, clear cell renal cell carcinoma

## Abstract

MicroRNAs (miRNAs) have emerged as critical modulators of carcinogenesis and tumor progression. In the present work, we sought to identify the biological function of miR-155 as well as its underlying mechanism in clear cell renal cell carcinoma (ccRCC). We examined the expression of miR-155 in clear cell RCC (ccRCC) and adjacent normal tissues and then explored the roles of miR-155 both *in vitro* and *in vivo*. The results of this analysis indicated that miR-155 activity was significantly upregulated in ccRCC tissues compared with the corresponding normal tissues. miR-155 was associated with ccRCC aggressiveness in both cell lines and clinical specimens, and a specific and inverse correlation between miR-155 and E2F2 expression was found in human ccRCC samples. Overexpression of miR-155 in 786-O cells decreased E2F2 expression while reduction of miR-155 by anti-miR-155 in ACHN cells elevated E2F2 expression. Re-expression of E2F2 in 786-O cells repressed the cell migration/invasion abilities elevated by miR-155, whereas knockdown of E2F2 in ACHN cells restored these cellular functions hampered by the miR-155 inhibitor. Using Western blot and luciferase reporter assays, we determined that E2F2 was a direct target of miR-155. Taken together, the *in vitro* and *in vivo* results demonstrate that miR-155 functions as a tumor-promoting miRNA by targeting E2F2 in ccRCC.

## INTRODUCTION

Renal cell carcinoma (RCC) is one of the most common urological malignancies in China over the years [1, 2]. The incidence of this disease has steadily increased. Undesired cell proliferation often results in the genesis and development of renal cell carcinoma. RCC is heterogeneous and comprises several histological subtypes classified according to differences in genetics, biology, and behavior [3]. Clear cell RCC (ccRCC), the most prevalent and aggressive RCC subtype, is characterized with extremely high rates of local invasion, malignancy, and mortality and resistance to chemotherapy and radiotherapy [4].

Recent advancements in ccRCC treatment, including targeting therapies, have been achieved and these approaches have changed the treatment landscape for patients with late-stage and metastatic RCC [5, 6]. Unfortunately, most treated patients eventually develop to the progressive disease because of acquired resistance, among other reasons [7, 8]. Hence, obtaining a better understanding of the mechanisms involved in the pathogenesis and progression of ccRCC and developing more effective therapeutic approaches are urgent endeavors.

MicroRNAs (miRNAs), a class of small non-coding RNAs measuring about 22 nucleotides in length, primarily function through interaction with the 3′untranslated region (3′UTR) of target mRNAs, resulting in inhibition of their translation or degradation [9, 10]. miRNAs are known to contribute to multiple tumorigenic steps in human cancers [11]. Recent studies have demonstrated the regulatory functions of miRNAs in ccRCC cell growth, apoptosis, migration and invasion [11–17]. Aberrant expression of miR-155 has been found in various cancers, including colorectal carcinoma, breast cancer and lymphoma [18–20]. However, the biological function and downstream targets of miR-155 in ccRCC remain largely unknown.

E2F transcription factor 2 (E2F2) is expressed at relatively low levels in ccRCC, where it is negatively correlated with miR-155 expression levels. E2F2 is a member of the E2F family, which plays imperative roles in proliferation, apoptosis, and several other biological activities [21, 22]. E2Fs bind to Rb protein, and the Rb/E2F complexes formed modulate the function of genes involved in the cell cycle, checkpoint control DNA synthesis, and apoptosis [23, 24]. The roles of E2F2 in ccRCC cell proliferation and invasion have not previously been investigated. In the present study, we demonstrate that miR-155 is significantly upregulated in ccRCC progression. Ectopic expression of miR-155 in cancer cells enhanced invasion *in vitro* and tumorigenic capacity *in vivo*. We identify and validate the E2F2 gene as a novel and direct target of miR-155, as assessed by mutagenic analysis of the 3′-UTR of the gene and its luciferase activity. We also discover, for the first time, that E2F2 acts as a tumor suppressor in ccRCC. Comprehensive studies indicate that E2F2 plays a critical role in miR-155-regulated cancer cell proliferation and invasion.

## RESULTS

### Distinguished miR-155 and E2F2 expressions in tumor and normal tissues of ccRCC

**Figure 1:**
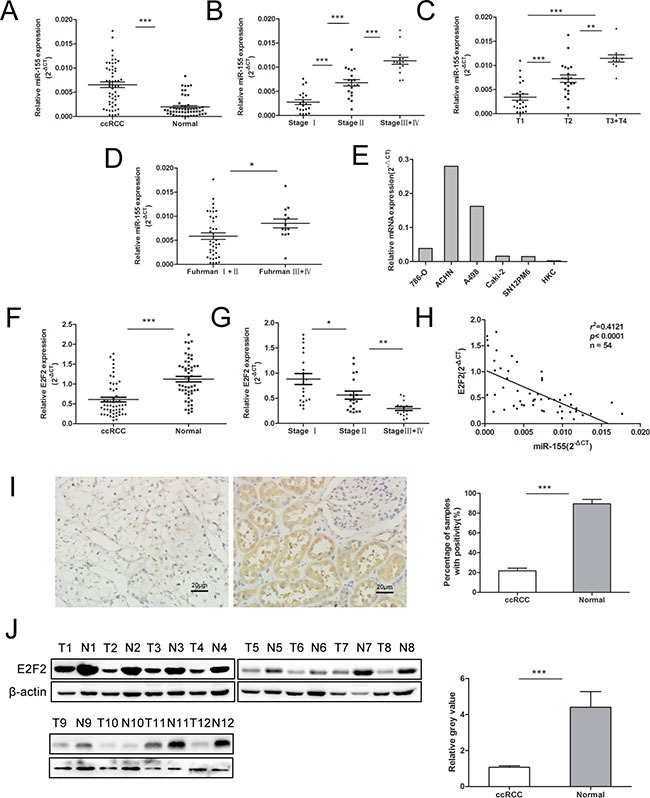
Expression of miR-155 in clinical samples and various cell lines and its relationship with E2F2 expression (**A**) miR-155 expression levels were significantly upregulated in ccRCC cancer tissues compared with normal tissues. (**B**–**D**) Correlations between miR-155 expression level and the clinical stage, T stage, Fuhrman grades of ccRCC respectively. (**E**) miR-155 expressions in various RCC cell lines compared with that in HKC. (**F**) Downregulation of E2F2 mRNA levels in ccRCC cancer tissues; here, normal tissues served as controls. (**G**) Correlations between E2F2 expression level and the clinical stage of ccRCC. (**H**) Negative correlation of E2F2 mRNA levels and miR-155 levels (*n* = 54, *r*^2^ = 0.4121, *P* < 0.0001). (**I**) Representative images of E2F2 IHC in ccRCC cancer tissues and their paired normal tissues. (**J**) Western blot of E2F2 showed alterations in protein levels consistent with variations in mRNA levels in clinical samples. Data represent the mean ± SD. (*P < 0.05; **P < 0.01; ***P < 0.001).

To understand the role of miR-155 in ccRCC, miR-155 expression was examined by qRT-PCR in several specimens (tumors and their corresponding normal tissue specimens) and a series of cell lines. Figure 1A reveals that miR-155 was significantly upregulated in ccRCC tumors compared with their corresponding normal tissues (*P* < 0.001). We then examined miR-155 expression in different stages of ccRCC. Upregulated miR-155 expression was found in more developed tumor stages (Figure 1B). Considering more clinicopathological parameters, miR-155 expression was identified to be elevated with T stages (*P* < 0.001) (Figure 1C) and Fuhrman grades (*P* < 0.05) (Figure 1D). Furthermore, miR-155 expression was measured in multiple cell lines with different malignant manners. Figure 1E reveals that miR-155 showed relatively highly expression in ACHN and A498 cells but relatively low expression in 786-O, Caki-2, and SN12PM6 cells. Interestingly, miR-155 was barely expressed in the HKC cell line. These results are in accordance with the miR-155 expression levels observed in the tissue specimens. At the mRNA level, E2F2 expression was significantly downregulated in ccRCC cancer tissues compared with normal tissues (*P* < 0.001) (Figure 1F). And the E2F2 expression was mitigated with development of clinical stages (Figure 1G). Moreover, miR-155 expression obviously showed an inverse relation to E2F2 expression at the mRNA level (*r*^2^ = 0.4121, *P* < 0.0001) (Figure 1H). To further identify the relationship between miR-155 and E2F2, we knockdown and overexpressed E2F2 *in vitro*, the level of miR-155 did not change with the alteration of E2F2 expressions (Supplementary Figure S1). To study the association between E2F2 expression and the clinicopathological factors of ccRCC, we examined E2F2 expression patterns in 102 primary ccRCC tissues and their corresponding normal tissues by immunohistochemistry (Figure 1I). Protein levels of the E2F2 were also assessed by Western blot (Figure 1J). The results obtained indicated that miR-155 acts as an oncogene in ccRCC progression and that E2F2 may serve as a tumor suppressor.

### Overexpression of miR-155 promotes ccRCC cell proliferation and motility *in vitro*

**Figure 2:**
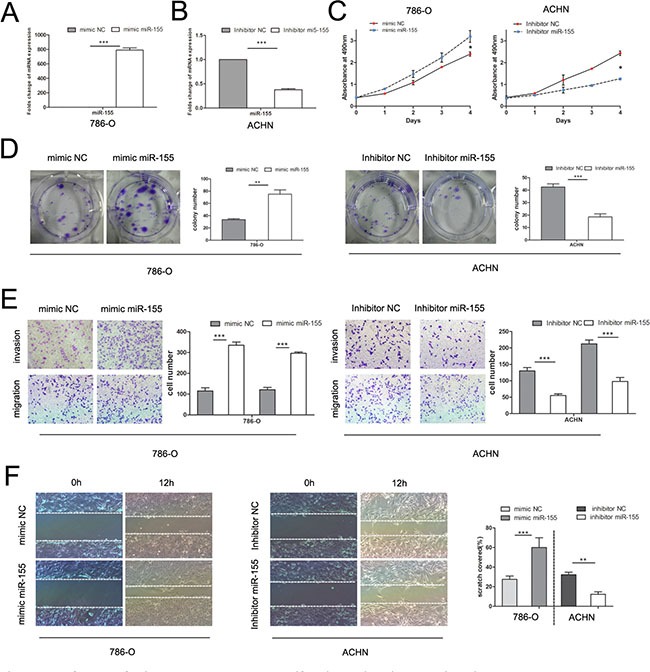
Influence of miR-155 on tumor cell proliferation, migration, and invasion (**A**) Alteration of the miR-155 expression levels of 786-O cells 48 h after transfection with the miR-155 or NC mimics. (**B**) Alteration of the miR-155 expression levels of ACHN cells 48 h after transfection with the miR-155 or NC inhibitors. (**C**) MTS assay showed that transfection of the miR-155 mimic can significantly increase the proliferation velocity of 786-O cells and that the miR-155 inhibitor can attenuate proliferation in ACHN cells. (**D**) Effect of miR-155 overexpression and suppression on the colony formation of 786-O and ACHN cells, respectively. The number of foci of > 50 cells was counted after 14 d. (**E**) Representative photographs of transwell assays (magnification, ×100) of 786-O and ACHN cells to determine the oncogenic function of miR-155. (**F**) Wound healing assay was performed to reveal that the miR-155 mimic increases the cell viability of 786-O cells. In ACHN cells, miR-155 inhibition hampered cell motility. Data represent the mean ± SD. (*P < 0.05; **P < 0.01; ***P < 0.001).

To verify the biological function of miR-155 in ccRCC, the miR-155 mimic (155 M) was used to elevate the expression level of miR-155 and the miR-155 inhibitor (155I) was used to attenuate miR-155 expression levels. 786-O cells with relatively low levels of miR-155 (Figure 1C) were transfected with the miR-155 mimic to achieve miR-155 overexpression; cells were also transfected with the miR-155 negative control (NC) mimic as a control (Figure 2A). ACHN with high miR-155 expression was treated with the miR-155 inhibitor (Figure 2B). After confirming the efficacy of transfection by qRT-PCR, above two cell lines with different groups individually were then applied for MTS assay. As indicated in Figure 2C, 786-O cells transfected with the miR-155 mimic showed remarkably enhanced growth relative to the NC mimic cells (*P* < 0.05). Conversely, the miR-155 inhibitor significantly inhibited the growth of ACHN cells compared with the inhibitor NC cells (*P* < 0.05).

In the colony formation assay, 786-O cells transfected with the miR-155 mimic showed significantly increased colony formation compared with cells transfected with the NC mimic; after transfection with the miR-155 inhibitor, ACHN cells showed significantly inhibited colony formation (Figure 2D). To investigate the effect of miR-155 on the migration and invasion of ccRCC cells, we performed transwell and wound healing assays. 786-O cells treated with the miR-155 mimic showed significant increases in migratory and invasive abilities compared with cells treated with the miR-155 NC mimic. After transfection with the miR-155 inhibitor, migration and invasion of ACHN cells were significantly inhibited (Figure 2E). In the wound healing assay, tumor cell motility was taken to reflect migratory ability. miR-155 augmented 786-O cell migration 12 h after scratching, and ACHN cells treated with the miR-155 inhibitor displayed decreased migratory capacity compared with inhibitor NC-treated cells (Figure 2F). These results suggest that miR-155 promotes the proliferation and invasion of ccRCC cells.

### E2F2 acts as a tumor suppressor in ccRCC

**Figure 3:**
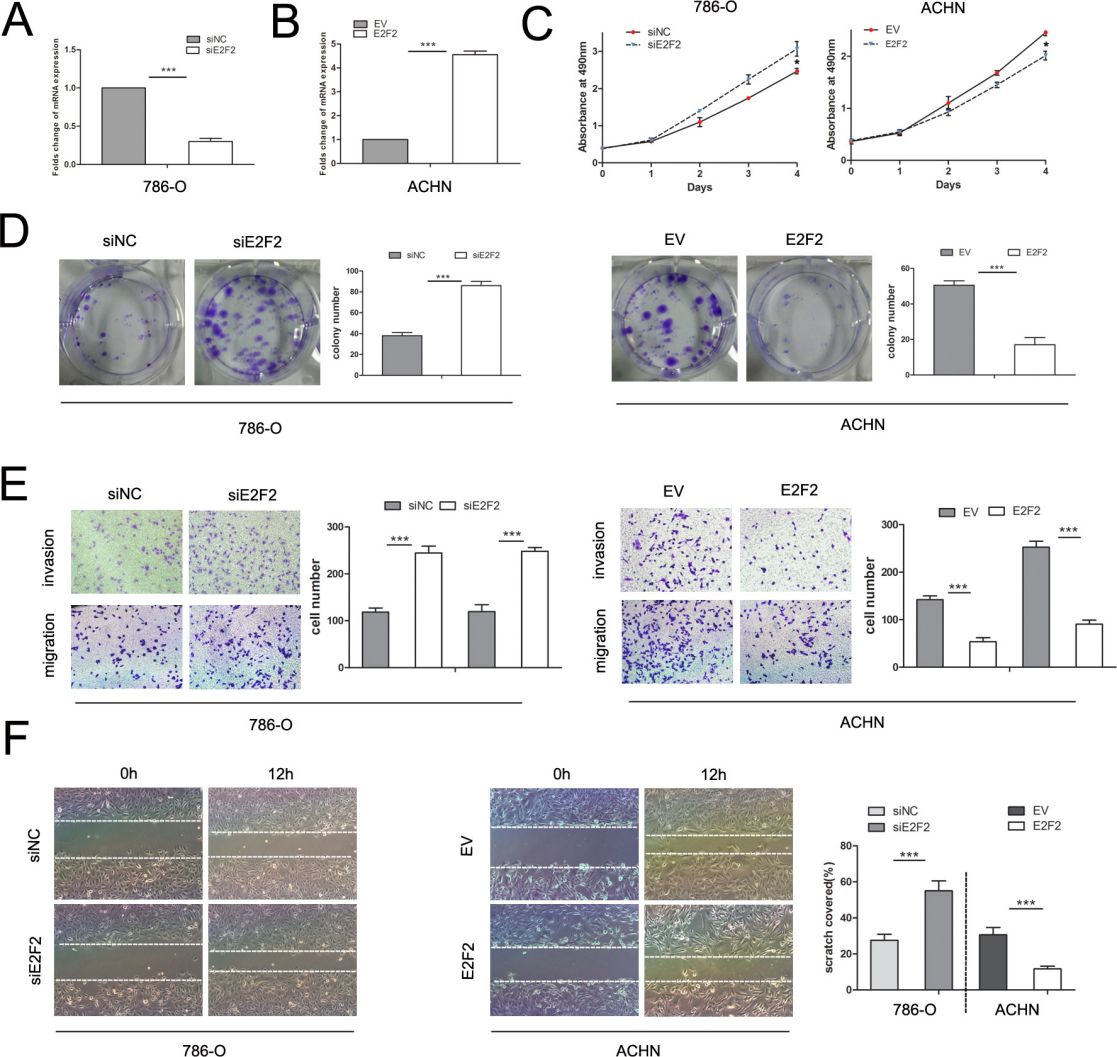
Effects of E2F2 on *in vitro* cell proliferation, motility, and migration (**A**) Alteration of E2F2 expression levels in 786-O cells 48 h after siRNA treatment compared with siNC. (**B**) Alteration of E2F2 expression levels in ACHN cells 48 h after transfection with lentiviral E2F2 plasmids compared with the empty vector. (**C**) MTS assay revealed that transfection of the siRNA of E2F2 significantly accelerated proliferation velocity in 786-O cells while E2F2 overexpression attenuated proliferation in ACHN cells. (**D**) Effect of E2F2 overexpression or suppression on the colony formation of ACHN and 786-O cells, respectively. The number of foci of > 50 cells was counted after 14 d. (**E**) Representative photographs of transwell assays (magnification, ×100) of 786-O and ACHN cells identifying E2F2 as a tumor suppressor. (**F**) E2F2 knockdown in 786-O cells significantly increased the number of viable cells; in ACHN cells, E2F2 overexpression largely decreased cell viability. Data represent the mean ± SD. Each experiment was performed in triplicate. (*P < 0.05; **P < 0.01; ***P < 0.001).

E2F2 plays an imperative role in cancer progression. To investigate its role in ccRCC, we employed the siRNA technique to knock down E2F2 and lentiviral particles to overexpress E2F2. RT-PCR and Western blot were used to detect the mRNA and protein expression levels of E2F2 after E2F2-siRNA transfection and lentiviral infection, respectively (Figure 3A and 3B). After transfection with E2F2-siRNA, proliferation of 786-O cells increased markedly compared with cells transfected with siNC (Figure 3C). ACHN cells infected with lentiviral-E2F2 particles presented boosted growth compared with empty vector-group cells. In colony formation assays, 786-O cells transfected with E2F2 siRNA showed significantly increased colony formation compared with cells transfected with siNC, and ACHN cells transfected with the E2F2 plasmid showed obviously inhibited colony formation (Figure 3D). In the transwell assay, 786-O cells bearing siE2F2 showed obviously increases (*P* < 0.001) in migration and invasion compared with siNC cells. Conversely, the invasive ability of ACHN was significantly inhibited after infection with the E2F2-packed lentivirus (*P* < 0.001) (Figure 3E). In the wound healing assay, knockdown of E2F2 expression in 786-O cells significantly accelerated cell migration 12 h after scratching compared with siNC-group cells, and ACHN cells harboring E2F2 revealed impaired migratory capacity compared with empty vector-group cells (*P* < 0.001) (Figure 3F). These results suggest that E2F2 impairs the proliferation and invasion of cancer cells, which means E2F2 acts as a tumor suppressor in ccRCC.

### E2F2 is a direct target of miR-155

**Figure 4:**
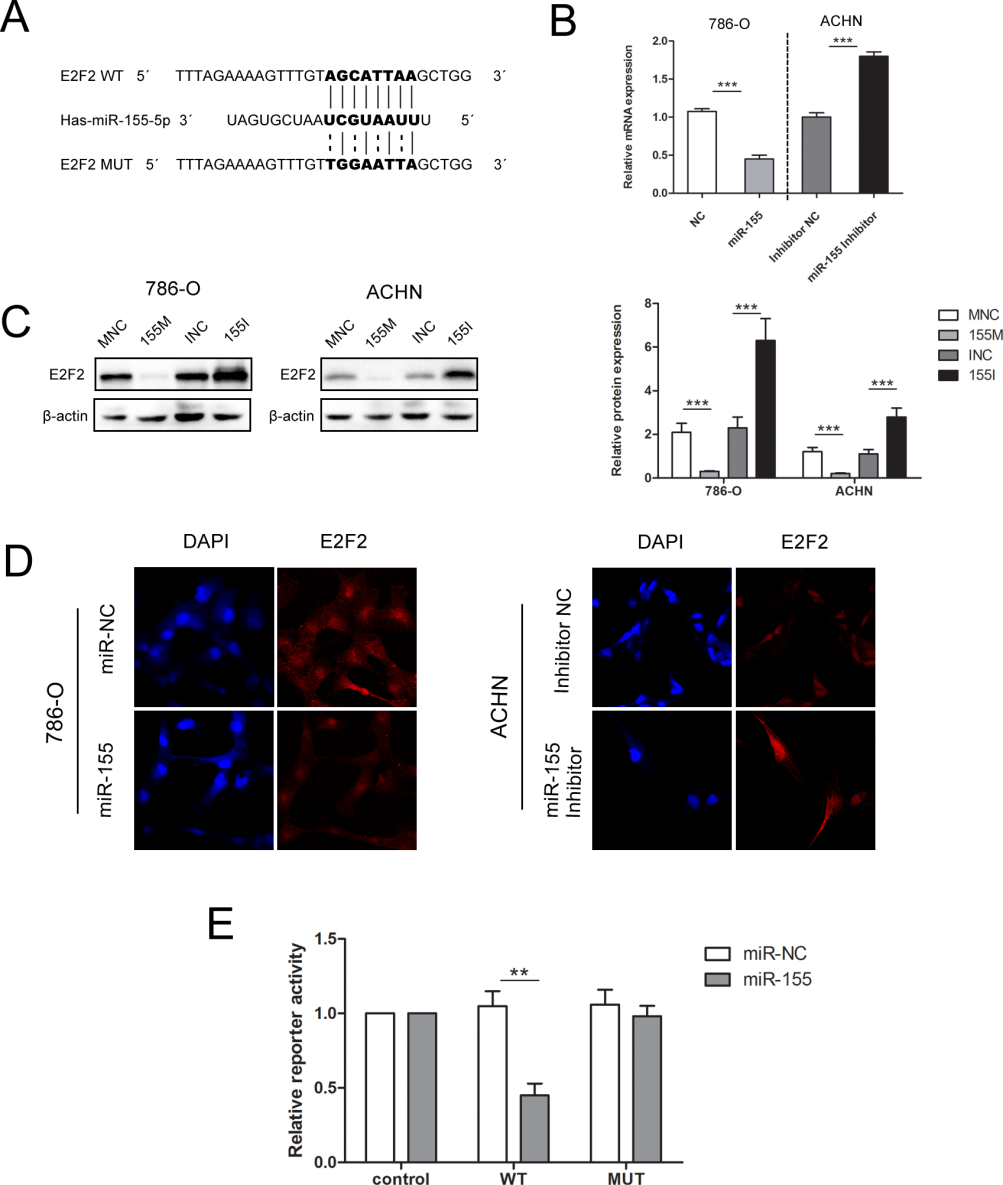
miR-155 downregulated E2F2 expression by specifically targeting its 3′UTR (**A**) Sequence alignment of the E2F2 3′UTR with wild-type (WT) versus mutant (MUT) potential miR-155 targeting sites. (**B**) mRNA levels of E2F2 were examined by qRT-PCR in 786-O and ACHN cells with different interferences. (**C**) Transfection of the miR-155 mimic or miR-155 inhibitor in 786-O and ACHN greatly changed E2F2 protein levels. (**D**) Immunofluorescence staining results showed the inverse effect of miR-155 on E2F2 in different cell lines. (**E**) Luciferase reporter assay showed decreased reporter activity after transfection of the wild-type E2F2 3′UTR reporter construct in 293T cells overexpressing miR-155. The E2F2 3′UTR MUT and control constructs showed no effect on reporter activity. Here, the *Renilla* luciferase construct was used as an internal control. The normalized luciferase activity of the control construct in each experiment was set to 1. Data represent the mean ± SD. (*P < 0.05; **P < 0.01; ***P < 0.001).

As E2F2 expression has been previously identified to be downregulated in ccRCC tissues and inversely associated with advanced disease stages, we hypothesized that high expression levels of miR-155 may induce RCC malignancy by downregulating E2F2 expression. RT-PCR and Western blot were performed to investigate the relationship between the expression levels of miR-155 and E2F2. As shown in Figure 4B and 4C, enforced miR-155 expression led to decreases in E2F2 mRNA and protein expression levels in both 786-O and ACHN cells; by contrast, application of miR-155 inhibitors to the 786-O and ACHN cell lines increased E2F2 expression.

In immunofluorescence assays, 786-O cells transfected with the miR-155 mimic (155 M) showed significantly decreased E2F2 protein levels compared with cells transfected with the miR-155 mimic NC (MNC). When ACHN cells were treated with the miR-155 inhibitor, E2F2 protein levels increased significantly compared with that in cells transfected with inhibitor NC (INC) (Figure 4D). These results indicate that protein levels of E2F2 are negatively regulated by miR-155.

Luciferase reporter assay was performed to explore the relationship between miR-155 and E2F2 in ccRCC. Bioinformatics predictions by miRDB (http://mirdb.org/miRDB/) and TargetScan (http://www.targetscan.org/) software validated the miR-155 binding site on the 3′UTR of E2F2 mRNA, and wild-type (WT) and mutant (MUT) luciferase reporter plasmids were constructed according to this site. To ascertain that E2F2 is a direct target of miR-155, a 403 bp fragment from the E2F2 3′UTR containing the putative miR-155 target site was cloned into psiCHECK2 plasmid. Dual-luciferase reporter assays were performed in 293T cells, which feature high transfection efficiency. miR-155 overexpression substantially repressed the activity of the reporter that carried the WT but not MUT 3′UTR of E2F2 (Figure 4E), thereby suggesting that regulation is mediated in a specific manner and that the predicted sequence is a miR-155 targeting site.

### miR-155-enhanced tumor proliferation and aggressive behavior is mediated by E2F2

**Figure 5:**
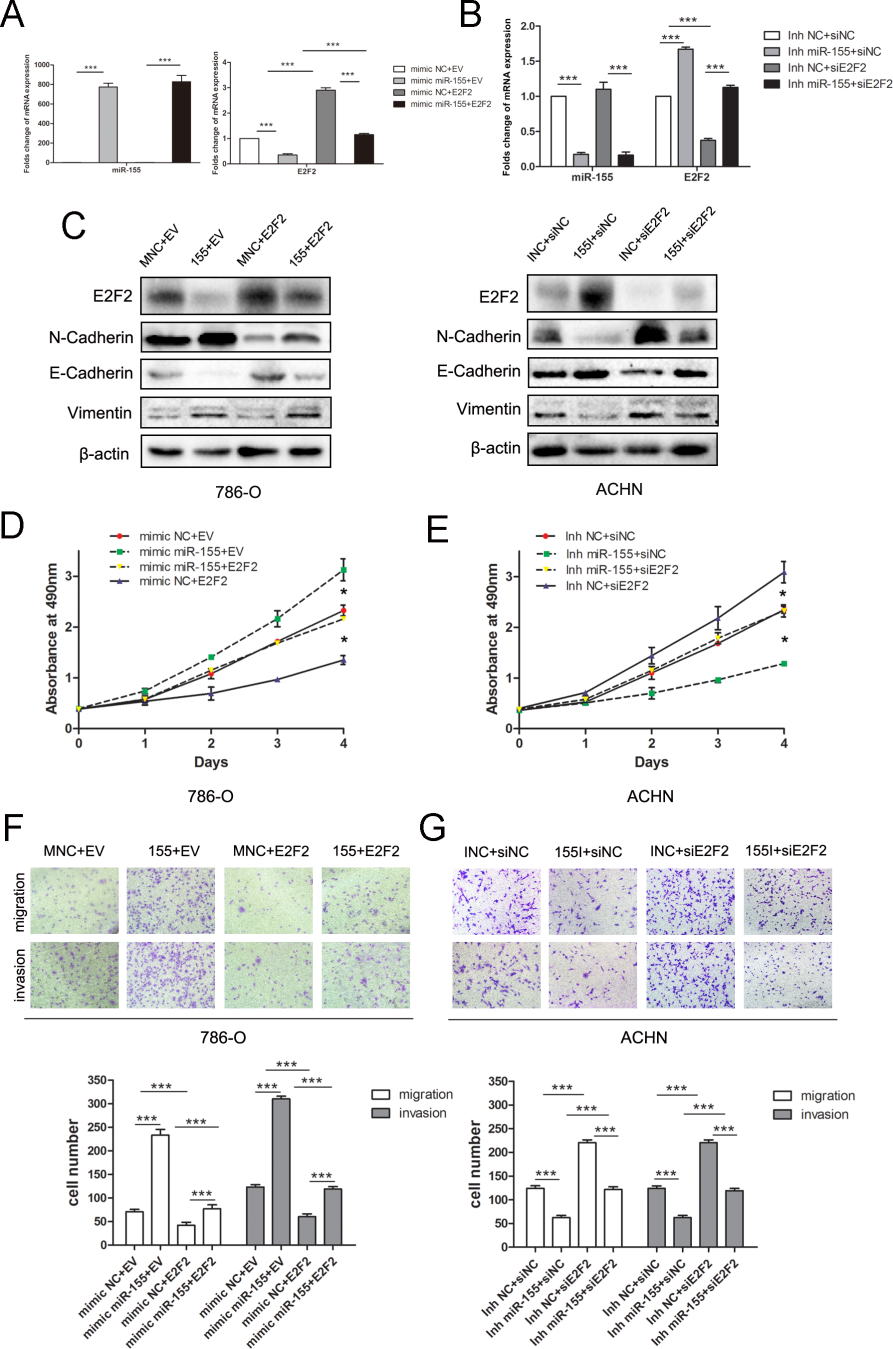
miR-155 regulated the proliferation, migration, and invason of ccRCC by targeting E2F2 (**A**) miR-155 and E2F2 mRNA level changes in 786-O cells with the miR-155 mimic (versus the control) and the E2F2 plasmid (versus the control) 48 h after transfection. (**B**) The mRNA levels of miR-155 and E2F2 in ACHN were analyzed by qRT-PCR after the use of miR-155 inhibitor and siE2F2. (**C**) E2F2 protein level alterations in 786-O cells with the miR-155 mimic (versus the control) and the E2F2 plasmid (versus the control) 48 h after transfection. After co-transfection of ACHN cells with siRNA duplexes (siE2F2 or the negative control) and miR-155 inhibitors (miR-155 or the negative control), the levels of E2F2 protein and EMT-related proteins were measured by Western blot. (**D**) E2F2 overexpression counteracted the positive proliferative effects of miR-155 in 786-O cells. (**E**) E2F2 inhibition hampered the negative proliferative function of miR-155 inhibitor in ACHN cells. (**F**) Cell migration and invasion in 786-O cells were analyzed by transwell assays. Cells were co-transfected with lentiviral particles (E2F2 or the empty vector) and miR-155 (mimic or the NC mimic). (**G**) Knockdown of E2F2 may reverse the effect of the miR-155 inhibitor on migration and invasion in ACHN cells. Data represent the mean ± SD. (*P < 0.05; **P < 0.01; ***P < 0.001).

We examined whether E2F2 overexpression could inhibit the oncogenic effects of miR-155 on ccRCC cell proliferation and progression. Lentiviral E2F2 particles and empty vector were introduced to 786-O cells that had been transfected with the miR-155 mimic and mimic NC. RT-PCR and Western blot analysis confirmed that the mimics markedly and specifically decreased E2F2 expression (Figure 5A). Transfection of the miR-155 inhibitor led to downregulation of miR-155 and upregulation of E2F2, as determined by qRT-PCR (Figure 5B) and Western blot. In addition, the EMT-related genes (N-Cadherin, E-Cadherin and Vimentin) were analyzed (Figure 5C). Proliferative and invasive abilities were impaired in 786-O/miR-155 cells after introduction of the E2F2 plasmid (Figure 5D and 5F). A rescue experiment was performed by co-transfecting E2F2 siRNA (versus the siNC) and the miR-155 inhibitor (versus the negative control) into ACHN cells. Attenuation of ACHN cell proliferation and invasion induced by the miR-155 inhibitor was effectively reversed by E2F2 downregulation (Figure 5E and 5G). Taken together, these findings clearly reveal that E2F2 is a direct and functional target of miR-155.

### miR-155 promotes tumor growth and cell invasion *in vivo*

**Figure 6:**
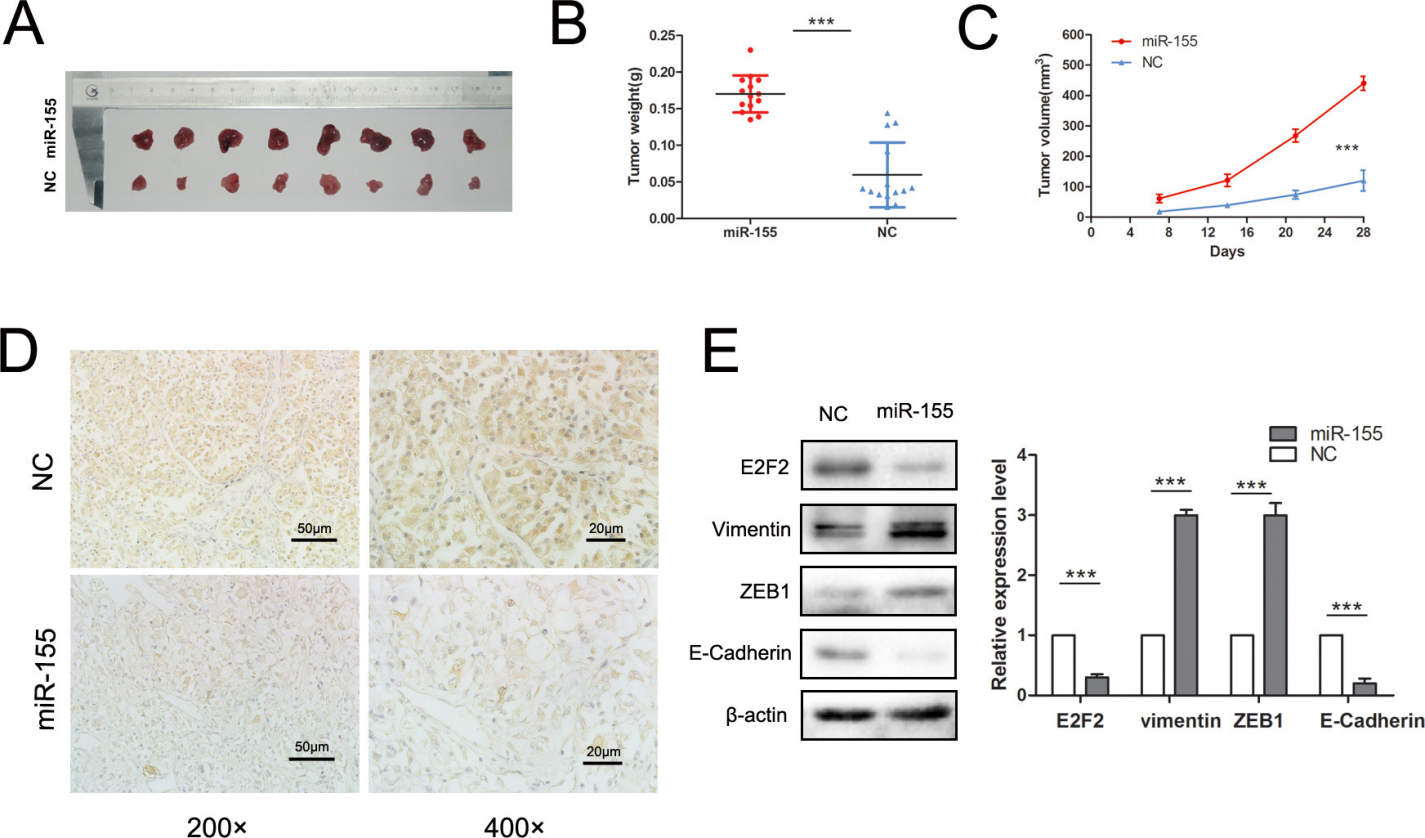
Overexpression miR-155 promoted tumor growth *in vivo* (**A**) Mouse tumors were obtained and dissected 4 weeks after subcutaneous injection of the transfected 786-O/miR-155 plasmid or 786-O/EV transfected cells. (**B**, **C**) Comparison of tumor weights and volumes between the 786-O/miR-155 and 786-O/EV groups. (**D**) Protein levels of E2F2 were immunohistochemically evaluated in the experimental and control groups. (**E**) EMT-related markers, including E-cadherin, ZEB1, and vimentin, were detected by Western blot between the groups. Data represent the mean ± SD. (*P < 0.05; **P < 0.01; ***P < 0.001).

An animal experiment was employed to verify the function of miR-155 in ccRCC aggressiveness. 786-O/miR-155 and 786-O/miR-NC cells were injected into nude mice subcutaneously. The results demonstrated that overexpression of miR-155 in 786-O cells boosted tumor growth and tumor size (*P* < 0.001) (Figure 6A–6C) compared with the controls. Mouse tumors were obtained and dissected 4 weeks after injection. As expected, the tumors formed from 786-O/miR-155 cells showed significantly low staining intensity of E2F2 relative to the tumors produced from 786-O/miR-NC cells (Figure 6D). Considering that the EMT process is crucial in acquiring malignancy during cancer progression and that overexpression of miR-155 *in vitro* and *in vivo* can alter cancer cell migration and invasion, the expressions of several EMT-related markers, including E-Cadherin, Vimentin and ZEB1, upon exposure to miR-155 were further examined. The expressions of Vimentin and ZEB1 were significantly enhanced (*P* < 0.001) whereas that of E-Cadherin was greatly decreased (*P* < 0.001) in 786-O/miR-155 cells compared with the corresponding observations in 786-O/miR-NC cells (Figure 6E). Hence, our *in vivo* study results indicate that miR-155 promotes the EMT process in ccRCC cells.

## DISCUSSION

Recent advances have revealed dysregulation of miRNAs as a common event in cancers. Modulation of miRNA expression has been proposed to be a rising feature in renal cancer. The results of genome-wide expression profiling of microRNAs using microarray analysis of matched cancer tissue samples and their corresponding normal tissues have identified dozens of overexpressed and downregulated microRNAs between groups [25–28]. The differential microRNA patterns observed indicate a solid basis for further elucidation and functional research. In particular, the observation of elevated miR-155 expression in many profiles has led our team to validate the roles of this miRNA.

Upregulation of miR-155 is a prevalent event in multiple types of cancers. miR-155 has been reported to correlate with diverse biological functions, including proliferation, migration, invasion, and cell cycle progression [29–32]. In nasopharyngeal carcinoma, the miR-155 inhibitor could inhibit cell migration by targeting ZDHHC2 [33]. Mechanistic studies have demonstrated that inhibition of miR-155 induced upregulation of SOCS1 expression and subsequent inhibition of STAT3 in various malignant events [34]. Li found that miR-155 exerted suppressive functions in proliferation and induced apoptosis by upregulating BACH1 in renal cancer cells [35]. In chordoma, miR-155 may independently predict specific outcomes [36]. Upregulation of miRNA-155 promoted tumor angiogenesis by targeting VHL and was associated with poor prognosis and triple-negative breast cancer [37]. Several reports have demonstrated that miR-155 is often correlated with poor prognosis and may act as a key regulator of colorectal carcinoma progression [38, 39]. A recent study also showed that miR-155 inhibited tumor suppressor DMTF1 expression in bladder cancer [40]. In summary, the ability of miR-155 to regulate the expression of multiple targets indicates its pivotal role in the regulatory network of cancer development.

The miR-155 target was predicted to explore the mechanism underlying the function of miR-155 in ccRCC. Our results established E2F2 as a direct effector of miR-155. E2F2 is a member of the E2F family, which exerts important functions in regulating cell proliferation by modulating downstream genes such as cyclin and cyclin-dependent kinases [41]. E2F1, E2F2, and E2F3 are considered transactivators in multiple biological processes; while they have certain similar functions, they also possess unique properties individually [42]. Previous data suggested that the proliferation-promoting E2F1 and, especially, E2F2 played pivotal roles in the tumor biology of ovarian cancer [43]. E2F1, E2F2, and E2F3 are responsible for the oncogenic transformative property of mouse embryonic fibroblasts. Inactivation of p21 recovered the ability of E2F1–3-deficient cells to go through the G1/S phase [44]. As an emerging initiator for carcinogenesis, aberrant E2F2 expression has been reported to be associated with cancer progression and metastasis. Reintroduction of E2F2 to Myc-overexpressing T cells resulted in promotion of apoptosis and inhibition of tumorigenesis [45].

Analysis showed a good inverse correlation between levels of miR-155 and E2F2 mRNA in ccRCC tissues (*r*^2^ = 0.4121, *P* < 0.0001). Specifically, lower miR-155 levels were associated with higher E2F2 mRNA expression and vice versa. The relationship between miR-155 and E2F2 has been well established. Overexpression of E2F2 in ccRCC cells attenuated cell growth and suppressed cell migration and invasion, similar to the phenotypic alterations observed upon miR-155 knockdown. In addition, *in vitro* studies showed that E2F2 had no effect on miR-155 expression. The results of luciferase reporter assay revealed that miR-155 could bind to the WT target sequence but not the MUT target sequence. Upregulation of miR-155 in ccRCC cells significantly downregulated E2F2 expression through direct interaction with the 3′UTR of E2F2. Restoration of E2F2 attenuated the promotive effects of miR-155 on RCC cell migration and invasion. By contrast, the siRNA of E2F2 rescued the suppressive effects of the miR-155 inhibitor on RCC cell migration and invasion. The EMT process is generally acknowledged to play a crucial role in cancer malignancy and metastasis [46]. An *in vivo* study revealed that E-Cadherin, Vimentin, and ZEB1 were remarkably changed miR-155 treatment. Further *in vivo* studies based on a nude mice model of 786-O cells revealed that overexpression of miR-155 led to pronounced increases in tumor volume and weight. These data clearly demonstrate that miR-155 plays an important role in ccRCC tumorigenesis and progression by negative regulation of its target E2F2; thus, miR-155 may be a potential therapeutic target for ccRCC treatment.

## MATERIALS AND METHODS

### Surgical specimens

Matched cancerous and normal tissues were obtained from patients with ccRCC in the Department of Urology, Chinese People's Liberation Army (PLA) General Hospital (Beijing, China), immediately snap-frozen in liquid nitrogen, and stored at −80°C for RNA and protein extraction. Informed consent was obtained from the patients, and the research procedure was approved by the Medical Ethics Committee of Chinese People's Liberation Army (PLA) General Hospital. All included RCC cases were clinically and pathologically identified to be of the clear cell type. Clinical stages and T stages were determined according to the 2011 Union for International Cancer Control (UICC) TNM classification guidelines.

### Cell lines and cell culture

Human renal carcinoma cell lines, including 786-O, ACHN, Caki-2, A498, and HKC, were preserved in our laboratory. The SN12PM6 cell line was kindly provided by Dr. X.P. Zhang of the Department of Urology, Union Hospital (Wuhan, China). To perform the *in vitro* functional studies, 786-O and ACHN cells were maintained in high-glucose Dulbecco's modified Eagle's medium (HyClone) supplemented with 10% fetal bovine serum (FBS) (Gibco) and MEM-EBSS (HyClone), respectively, as well as penicillin (100 U/mL) and streptomycin (100 U/mL). Both cell lines were cultivated in a humidified incubator maintained at 37°C with 5% CO_2_.

### RNAi treatment

The miR-155 mimic, inhibitor, and related negative controls (NCs) were designed and synthesized by Genepharma (Shanghai, China). Short interfering RNAs (siRNAs) against E2F2 (siE2F2) and the negative control (siNC) with non-functioning sequences were also synthesized by Genepharma. The sequences of the E2F2 siRNA are as follows: 5′-CCUACUACACACCGCUGUATT-3′ (sense) and 5′-UACAGCGGUGUGUAGUAGGTT-3′ (antisense). The sequences of siNC are as follows: 5′-UUCUCCGAACGUGUCACGUTT-3′ (sense) and 5′-ACGUGACACGUUCGGAGAATT-3′ (antisense). Lipofectamine 2000 (Invitrogen, Carlsbad, CA) was used for transfection according to the manufacturer's protocol. Cells were used for the necessary experiments 48 h after siRNA transfection.

### Total RNA extraction and quantitative real time PCR (qRT-PCR)

The total RNA of tissues and cells was extracted with Trizol reagent (Invitrogen). Reverse transcription of miRNA and mRNA was performed using a miRcute miRNA first-strand cDNA kit (Tiangen, China) and one-step RT-PCR kit (TransGen, China) according to the manufacturers' instructions. miR-155 expression levels were detected via the SYBR Green qRT-PCR method by using an Hsa-miR-155 miRcute miRNA qPCR detection kit (Tiangen); here, miR-155 expression was normalized to U6 snRNA and the relative expression of miR-155 was calculated using the power formula: 2^−Δ^Ct (ΔCt = Ct miR-155-Ct U6). E2F2 mRNA expression was detected by using an Applied Biosystems 7500 Detection system with SYBR Green (TransGen); here, the relative mRNA levels of genes were normalized to peptidylprolyl isomerase A (PPIA) using the 2^−Δ^CT method. The primers of E2F2 are as follows: 5′-AAGTGCATCAGAGTGGAT-3′ (sense) and 5′-AGTGTCATACCGAGTCTTC-3′ (antisense). The primer sequences of PPIA are as follows: 5′-ATGGTCAACCCCACCGTGT-3′ (sense) and 5′-TCTGCTGTCTTTGGGACCTTGTC-3′ (antisense). All experiments were repeated three times and each sample was tested in triplicate.

### Western blotting

Cells were harvested 48 h after transfection and prepared for lysis in RIPA. Protein concentration was tested using the BCA method. The separated proteins were transferred to a PVDF membrane (Millipore). Membranes were blocked and then incubated with primary antibodies against E2F2 (Santa Cruz, sc-632, USA), N-Cadherin (Abcam, Ab76057), E-Cadherin (Cell Signaling Technology, #3195, USA), Vimentin (Cell Signaling Technology, #5741, USA), ZEB1 (Cell Signaling Technology#3396, USA), or β-actin (ZSGB-BIO, TA-09, China) followed by incubation with horseradish peroxidase-conjugated secondary antibodies. Protein expression levels were normalized to β-actin, and the detailed procedure was performed as previously reported [47].

### Construction of plasmids

The 3′-UTR of E2F2 containing the miR-155 binding site was amplified by PCR using its specific forward primer with the Xho1 restriction site (5′-CCGCTCGAGGGCTATGACTTCTGGA-3′) and reverse primer with the Not1 site (5′-ATAAGAATGCGGCCGCTTGTGAGTATTT-3′). The PCR product was digested with Xho1/Not1 and inserted into psiCHECK2, after which the mutation plasmid was generated by Genewiz (Beijing, China). All constructs were verified by sequencing. During plasmid construction, the coding domain sequence of E2F2 was amplified from pcDNA3.1-E2F2 by high-fidelity PCR amplification. The resulting fragment was inserted into the lentiviral vector PLV–EGFP(2A) Puro (Inovogen Tech. Co.) between XbaI and EcoRI to generate PLV–EGFP-E2F2. The required sequence was confirmed by DNA sequencing. E2F2 expression was evaluated by real-time reverse transcription PCR (RT-PCR) and Western blot. The miRNA-155(NR_030784.1) segment was cloned into pLVshRNA-EGFP(2A)puro to establish a stable transfected cell line.

### Luciferase activity assay

293T cells at 60% confluence were seeded into 6-well plates and then transfected with the luciferase reporter gene construct (WT and MUT) in combination with miR-155 mimic (GenePharma) or the negative control miR-NC. Transfection was performed with Lipofectamine2000 (Invitrogen). Luciferase activity was measured using a Dual Luciferase Reporter Assay kit (Promega). Firefly luciferase activity was normalized to *Renilla* luciferase activity for each transfected well. Data are expressed as the mean ± SD. The luciferase activities of Firefly (F) and *Renilla* (R) were measured 48 h after transfection, and the relative luciferase activity was expressed as F/R. All transfection experiments were conducted in triplicate and repeated thrice independently.

### Immunohistochemistry (IHC)

A standard immunostaining procedure was performed as previously reported [48]. A primary antibody against human E2F2 (1:100 dilution) was applied to human ccRCC cancer tissues and their corresponding normal tissues. Slides generated from the *in vivo* study were also stained with E2F2 antibody. Immunostaining for E2F2 protein was independently and blindly analyzed by two pathologists.

### MTS assay

Cell proliferation and viability was tested by absorbance using the MTS assay. After transfection, 1000 cells of each sample were plated on a 96-well plate in triplicate. Cell viability was assessed at 24, 48, 72, or 96 h after treatment as previously described using an automatic enzyme-linked immunosorbent assay reader (BioTek Instruments) [47].

### Colony formation assay

Cancer cells were plated on 6-well plates and transfected with the indicated miR-155 mimic, miR-155 inhibitor, E2F2 plasmid, or siRNAs. Exactly 48 h later, the cells were collected, seeded into 6-well culture plates. Colonies consisting of at least 50 cells were counted 14 days later.

### Migratory and invasion assays

A 24-well transwell plate with 8 μm pore polycarbonate membrane inserts (Corning Costar Corp., Cambridge, MA) was employed to assess the migration and invasive capacity of cells according to the manufacturer's protocol. For the invasion assay, the membrane was pre-coated with Matrigel (BD Biosciences, Bedford, MA). After 4 or 8 h of incubation, cells invading into the lower surface of the membrane were fixed and stained with methanol mixed with crystal violet and then counted under a microscope. All assays were performed in three independent experiments.

### Wound healing assay

For the wound healing assay, 786-O and ACHN cells were seeded on 6-well plates with fresh medium containing 10% FBS. After formation of a confluent monolayer of cells, the membrane was scratched using a sterile 200 μL pipette. The cell culture medium was replaced and photographs of the wound were taken at different time points (0 and 12 h after scratching). The coverage of the intermediate space was measured at three positions for each replicate, and experiments were performed in triplicate.

### *In vivo* tumor growth assay

All experimental procedures involving animals were performed according to guidelines for the care and use of laboratory animals and institutional ethical guidelines for animal experiments. 786-O/miR-155 or 786-O/miR-NC cells (5 × 10^6^ in 0.1 mL of sterilized saline) were implanted subcutaneously into the left armpit of 4–5-week-old male nude mice (14 mice per group). Tumor volume was measured in three dimensions (a, b, c) every one week using calipers and calculated as abc × 0.52. Twenty-eight days after implantation, the animals were sacrificed. Tumors were dissected and sectioned; one section was fixed in formalin and embedded in paraffin and another section was frozen in liquid nitrogen and stored at −80°C for Western blot analysis. Expressions of E2F2 and EMT-markers were examined. The animal studies and experimental protocol were approved by the Institutional Animal Care and Use Committee of the Chinese PLA General Hospital.

### Statistical analysis

Data are presented as the mean ± SD of three independent experiments. All data were analyzed using SPSS statistical software version 18.0 (SPSS Inc., Chicago, IL), and *P* < 0.05 was considered statistically significant. Correlations between two variables were analyzed by linear regression. Continuous data were examined for normality by using the Kolmogorov-Smirnov test. Student *t*-test or one-way ANOVA was applied to compare normally distributed variables. The Mann-Whitney U or Kruskal-Wallis test was used to compare continuous variables not adapting to the assumptions of normality.

## SUPPLEMENTARY MATERIALS FIGURE


