# Supplementary material for: miR-155 regulates the proliferation and invasion of clear cell renal cell carcinoma cells by targeting E2F2
Source: Oncotarget. 2016 Mar 7;7(15):20324–37. doi: 10.18632/oncotarget.7951 (PMC4991458; doi:10.18632/oncotarget.7951)
Supplement: Supplementary file 1 [file oncotarget-07-20324-s001.pdf]

miR-155 regulates the proliferation and invasion of clear cell renal cell carcinoma cells by targeting E2F2

Supplementary Materials

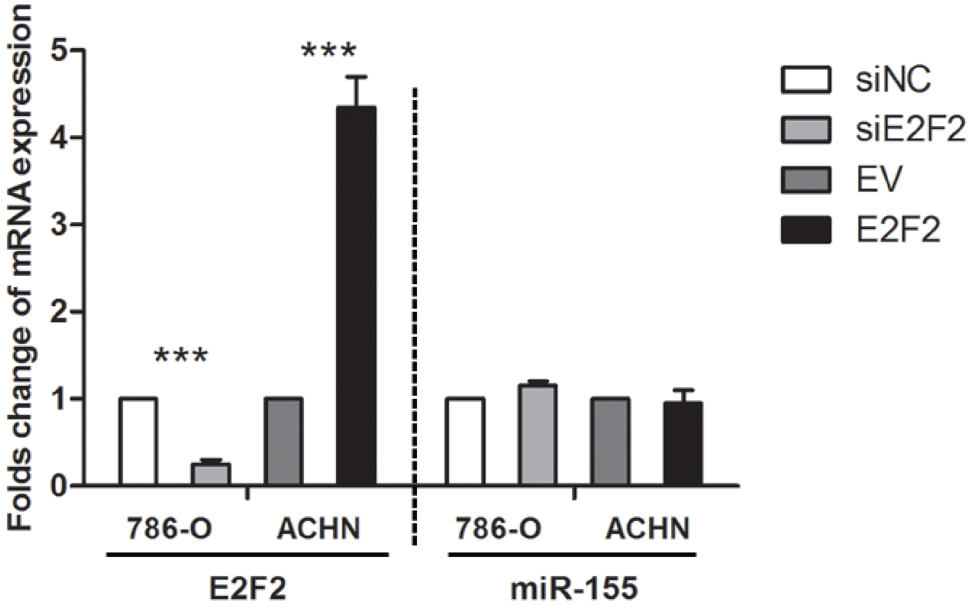

Supplementary Figure S1.
